# Harnessing Artificial Intelligence for Advancing Medical Manuscript Composition: Applications and Ethical Considerations

**DOI:** 10.7759/cureus.71744

**Published:** 2024-10-17

**Authors:** Shruti Singh, Rajesh Kumar, Vikas Maharshi, Prashant K Singh, Veena Kumari, Meenakshi Tiwari, Divya Harsha

**Affiliations:** 1 Pharmacology, All India Institute of Medical Sciences, Patna, Patna, IND; 2 General Surgery, All India Institute of Medical Sciences, Patna, Patna, IND; 3 Plastic Surgery, All India Institute of Medical Sciences, Patna, Patna, IND; 4 Lab Medicine, All India Institute of Medical Sciences, Patna, Patna, IND

**Keywords:** ai technologies, applications, artificial intelligence, ethical issues, medical manuscript writing

## Abstract

Scientific medical manuscripts are fundamental to advancing research and enhancing patient care. With the emergence of artificial intelligence (AI), the process of composing such manuscripts has witnessed profound transformations. This review delves into the multifaceted role of AI in medical manuscript composition, analyzing its applications, benefits, drawbacks, and ethical implications. Employing a comprehensive narrative review methodology, we explored databases such as PubMed, Google Scholar, and Science Direct. The review charts the evolution of AI in medical writing, from basic word processing to sophisticated neural network-based models like GPT-3 and GPT-4. Various AI-powered tools such as ChatGPT, Google Bard, Elicit, and Consensus AI are examined in terms of their functionalities and contributions to research and medical writing. While AI technologies offer notable advantages in automating content creation and boosting research productivity, concerns persist regarding overreliance, potential homogenization of writing styles, and ethical considerations such as originality and authorship. Because of this concern, some companies are restricting the use of AI in peer review processes, medical examinations, etc. It is crucial to strike a balance in integrating AI tools, ensuring human oversight, conducting thorough algorithm audits, addressing financial implications, and upholding academic integrity. The review underscores the transformative potential of AI in medical manuscript composition while emphasizing the ongoing significance of human expertise, creativity, and ethical responsibility in scientific communication. Recommendations are provided for the effective integration of AI tools into medical writing processes, emphasizing collaborative efforts between AI developers, researchers, and journal editors to navigate ethical dilemmas and maximize the benefits of AI-driven advancements in scientific publishing.

## Introduction and background

Introduction

The composition of scientific medical manuscripts is a fundamental component of the medical research field, serving as the primary medium through which breakthroughs, insights, and findings are communicated within the scientific community and beyond. This process demands a high level of scientific understanding, clarity of expression, and strict adherence to ethical standards and regulatory guidelines. Efficiency, accuracy, and compliance are pivotal in ensuring that these manuscripts contribute effectively to the body of medical knowledge, facilitating timely peer review and publication processes, and ultimately supporting healthcare advancements and patient care [1-3].

The advent of artificial intelligence (AI) has introduced a transformative potential for enhancing the writing process of scientific medical manuscripts. AI technologies, particularly those leveraging natural language processing (NLP) and machine learning (ML), offer promising tools to automate and refine various aspects of manuscript preparation. From conducting comprehensive literature reviews to ensuring consistency in data interpretation and reporting, AI can significantly streamline the drafting process, improve the precision of scientific communication, and ensure compliance with intricate publication standards.

However, integrating AI into the manuscript composition process has its challenges. Concerns regarding data security, the potential loss of authorial voice, and the risk of overreliance on technology for critical thinking and creativity pose significant considerations. Moreover, the ethical implications of AI-assisted research documentation, including authorship and accountability, warrant careful deliberation [4].

This review aims to explore the multifaceted role of AI in the composition of scientific medical manuscripts. By examining its applications, benefits, and limitations, we seek to provide a comprehensive overview of how the commonly used AI technologies are currently being utilized in the field, their potential to revolutionize medical research communication, and the challenges that need to be addressed to maximize their positive impact on scientific discovery and dissemination.

Objectives

This review aims to trace the evolution of AI in medical writing, highlighting the significant milestones and developments that have shaped its current role. It will also examine the present-day applications of AI-powered tools, exploring their benefits, limitations, and the ethical considerations associated with their use in the medical writing field. Furthermore, the review will offer recommendations for the effective integration of AI tools to enhance productivity and accuracy in medical writing. Finally, it will outline potential future directions and advancements in AI technologies, identifying areas where further innovation could lead to improved outcomes in the medical writing industry.

## Review

Methodology

In composing our narrative review centered on the role of AI in research and medical manuscript preparation, we rigorously adhered to established protocols for such scholarly works, ensuring methodological thoroughness and trustworthiness. We undertook an exhaustive search across prominent databases, including PubMed, Google Scholar, and Science Direct. The search was guided by a carefully selected set of terms such as "AI in healthcare communication," "natural language processing," "machine learning applications," "scientific manuscript composition," and "AI ethical implications in scholarly publishing." The scope of our review was confined to comprehensive articles written in English that comprised authentic and relevant journals, educational institutional libraries, technology and tool websites for specific AI tools, blogs and commentaries, research databases and repositories, observational studies, and prior narrative reviews. Items such as letters to the editor and brief communications were excluded if they did not shed light on factors influencing the use of AI in medical writing. Ethical committee clearance was not applicable to this review since it involved collating and analyzing existing published literature rather than generating new data from living subjects.

Evolution of AI in medical writing

The history and current state of word processing AI and AI-assisted writing have evolved significantly over time, driven by advancements in NLP and ML. Initially focusing on basic editing and formatting in the 1980s-1990s, word processing technology advanced to include grammar checkers in the 1990s-2000s, utilizing rule-based algorithms. The 2000s-2010s introduced predictive text capabilities with statistical models to improve writing efficiency. The 2010s-2020s saw a leap in technology with deep learning, leading to neural network-based models like GPT-3 and GPT-4, which could generate coherent and natural text for various applications, from grammar checking to creative writing assistance.

Currently, AI in writing encompasses a range of technologies, including natural language generation systems for automatic text generation, ML-based models for content creation, and neural network-based models like GPT and Bidirectional Encoder Representations from Transformers for high-quality text generation. Rule-based systems focus on structured output, while hybrid models combine various methods for enhanced accuracy and naturalness. AI-assisted writing tools now offer extensive support to writers by providing suggestions, grammar checks, and more, with ChatGPT representing the latest in AI writing assistance, capable of conversational responses and various NLP tasks. This evolution marks a shift toward more sophisticated and versatile tools, with AI increasingly taking on complex writing tasks, thus transforming the writing process and the role of writers [5-12].

Current applications, specific advantages, and disadvantages of the commonest AI-powered tools in research and medical writing

*ChatGPT*​​

ChatGPT is an advanced language generation model developed by OpenAI, designed to produce human-like text based on the input it receives. In medical writing, ChatGPT serves as a powerful tool for automating content creation, enhancing research productivity, and providing support in drafting accurate medical documents, thereby revolutionizing the way medical professionals and writers approach the creation and verification of medical content (Table 1) [13-20].

**Table 1:** Applications, specific advantages, and disadvantages of the use of ChatGPT in medical research and writing

S. No.	Applications
1.	Knowledge gap identification: assists in identifying areas lacking in research and formulating hypotheses
2.	Title generation: suggests potential titles for research articles
3.	Research development: aids in developing and refining the research question, problem statement, novelty, review of literature, methodology, results, discussion, limitations, strengths, etc. for all kinds of scientific papers
4.	Writing support: helps in composing case reports, original articles, review articles, meta-analysis papers, protocols, grant proposals, ethical reviews, critical appraisals, feedback, correspondence emails, answers to reviewers’ regulatory submission documents, etc.
5.	Statistical analysis: provides basic statistical analysis, interprets results, generates tables, prepares Excel sheets, and refines discussion points
6.	Source information discovery: finds relevant sources for academic writing tasks
7.	Article outlining: creates structured outlines for research articles and drafts initial versions
8.	Paraphrasing and writing: rewrites text for clarity and originality
9.	Summarization: condenses text within articles to highlight key points and insights
10.	Citation formatting: formats references and citations according to academic standards
11.	Plagiarism detection: identifies instances of potential plagiarism in written work
Advantages	
1.	Generating content: ChatGPT has achieved state-of-the-art performance in various natural language processing tasks, making it capable of generating coherent, informative, and congruent responses
2.	Brainstorming support: ChatGPT can assist in brainstorming ideas, suggesting topics, and exploring different angles for research papers
Disadvantages	
1.	Lack of originality: ChatGPT generates text based on patterns in its training data and does not generate original ideas, which can raise the risk of plagiarism in research papers
2.	Incorrect response and lack of expertise: ChatGPT may provide incorrect or inaccurate information due to not being an expert in scientific fields, leading to potential errors in research papers
3.	Lack of citations: Chat GPT may not cite sources, requiring human oversight for ethical standards and ensuring accuracy

Gemini (Google Bard)

ChatGPT and Google Bard, two pioneering AI tools in the realm of healthcare manuscript writing, share a core similarity in their mission to streamline the research and writing process, offering innovative solutions for synthesizing complex medical data into coherent, high-quality scientific documents. However, when comparing ChatGPT and Google Bard, especially in the context of manuscript writing in healthcare, several key differences emerge that could influence their utility and effectiveness in this specialized field (Table 2) [21-26].

**Table 2:** Differences between ChatGPT and Google Bard in the context of manuscript writing

Feature	ChatGPT	Gemini (Google Bard)
Source and timing of information	Training data ends in 2021, potentially limiting the usefulness of new research	Draws information from the internet, accessing the latest research and information. It has access to massive data
User experience	Effective for generating and summarizing text but may require more steps to modify responses	Designed to create more detailed responses and allows for easier modifications to questions and responses
Research and writing quality	Recognized for superior writing capabilities, especially for generating content based on provided data	Tailored for research, providing more relevant and current information crucial for healthcare manuscript writing
Personalized experience	Can personalize search results based on user's interests and preferences, potentially making it easier for users to find the information they seek quickly	Does not personalize search results based on the user's interests or search history; responses are tailored only to the specific input received during each interaction
Privacy and data storage	Stores user interactions; specific details on privacy settings may vary	Offers settings to manage data storage duration within Google account settings, addressing concerns about privacy and confidentiality
Plugins	Numerous plugins are available that provide additional use cases with various apps	Fewer number of plugins
Fine-tuning techniques	ChatGPT is only fine-tuned using supervised learning techniques	Google Bard is fine-tuned using both supervised and reinforcement learning techniques

In summary, Google Bard's strength lies in its research capabilities and access to current information, making it potentially more suitable for the initial stages of healthcare manuscript preparation. ChatGPT's strengths in content generation and summarization could make it more useful for drafting and refining manuscript sections. Researchers might benefit from using both tools at different stages of their writing process, depending on the specific requirements of their manuscript.

*Elicit*​​​​​​

Elicit is an innovative online tool developed by Ought, a non-profit ML research lab based in the United States. It serves as a free AI research assistant designed to automate parts of researchers' workflows, particularly in evidence synthesis and text extraction. By leveraging language models, Elicit streamlines the literature review process, pulling publications from Semantic Scholar to quickly provide researchers with relevant papers, summaries, and key information extraction into a research matrix. This tool is particularly useful for questions with empirical research in areas such as biomedicine, where it can identify interventions, randomized controlled trials, and structure inquiries like "What are the effects of x on y?" (Table 3) [27-29].

**Table 3:** Applications, specific advantages, and disadvantages of the use of Elicit in medical research and writing

S. No.	Applications
1.	Semantic similarity searches: Elicit uses semantic similarity to find papers related to a researcher's question, even if they don't use the same keywords, combined with keyword filters
2.	Custom summaries generation: the tool generates custom summaries (abstract summarization) for every search result based on the query, providing key information about the papers in an easy-to-use table
3.	Citation Graph Exploration: Elicit automatically searches forward and backward in the citation graph when researchers star results, finding more relevant papers to explore
4.	Customizable information display: researchers can customize what information they see about each paper through adding columns and filtering based on study type
5.	Idea brainstorming and research direction: Elicit is valuable for researchers looking for initial leads (idea brainstorming), defining research directions, and finding papers to cite in their work
Advantages	
1.	Improved search capabilities: Elicit leverages natural language processing models to search for papers without relying solely on perfect keyword matches, enabling a broader and more comprehensive search
2.	Dynamic summarization: Elicit's ability to summarize and extract information from research papers quickly can provide researchers with a broader understanding of a topic and access to relevant insights
3.	Insightful AI analysis: delivers abstract summaries, tests conducted, trustworthiness assessments, critiques, and other citations, enhancing understanding and critical evaluation of papers
4.	Quality indicators: integrates Scimago Journal & Country Rank to help quantify the prestige of journals, providing researchers with insights into the quality of their sources
5.	Search term Suggestions: suggests synonymous and related terms, aiding in comprehensive and effective literature searches
6.	Elicit is currently free to use after the researcher creates an account
Disadvantages	
1.	Limited database: relies only on publications in Semantic Scholar, excluding licensed journals or content behind paywalls, which may result in gaps in literature retrieval
2.	Limitations of language models: Elicit's performance and accuracy are dependent on the underlying language models and their limitations
3.	Question-driven search limitation: encourages full research questions for search, which may not suit all research workflows that rely on keyword-based searches with specific syntax or controlled vocabulary

Consensus

Consensus AI is an AI-driven search engine that enhances academic research by efficiently mining through over 200 million scientific documents to deliver relevant, evidence-based insights for any research question. It uniquely accesses peer-reviewed studies, making it a trustworthy resource for scholars. In manuscript writing, it aids by simplifying literature reviews, summarizing findings, and identifying research gaps, thereby enriching the research process and supporting the development of well-informed, thoroughly researched manuscripts (Table 4) [30-32].

**Table 4:** Applications, specific advantages, and disadvantages of the use of consensus in medical research and writing

S. No.	Applications
1.	AI-driven search engine: extraction of key findings quickly and directly from peer-reviewed scientific research papers
Advantages	
1.	Evidence-based answers: consensus utilizes AI to extract and distill findings directly from scientific research, providing rigorous and reliable information for informed decision-making
2.	Ad-free and unbiased: consensus presents results from scientists without the influence of marketing teams, providing unbiased information
3.	Reliable scientific sources and quality control: consensus focuses on peer-reviewed, published sources, ensuring that the information provided is based on credible scientific research
Disadvantages	
1.	Limited scope: consensus focuses on peer-reviewed, published sources, which may not cover all relevant research or include emerging studies that are yet to be published
2.	Limited accessibility: the availability and accessibility of research papers in Consensus may be restricted to those that are publicly available or require subscription access
3.	Potential lack of freshness: consensus may not always capture the most up-to-date research findings, as it relies on existing published literature

Elicit and Consensus serve distinct purposes within academic research, leveraging AI to aid users effectively. Elicit is tailored for deep research, offering analytical tools such as paper summarization, theme synthesis, and a unique feature for uploading and analyzing personal PDFs, making it particularly suitable for researchers needing detailed insights. Consensus, with more community upvotes, is designed for broader audiences seeking quick, reliable answers from scientific literature. It simplifies accessing evidence-based research through AI-summarized insights and supports personalized library creation. Despite their shared freemium model, Elicit stands out for in-depth analysis with technologies like Next.js and React, whereas Consensus focuses on efficiency and user-friendly access to research findings.

Research Rabbit 

Research Rabbit is an advanced digital tool designed to enhance the efficiency and depth of academic research, particularly aiding in manuscript writing. Leveraging AI, including NLP and ML, it analyzes the context of a user's research to recommend related academic papers and sources. This mechanism helps uncover relevant literature, fill knowledge gaps, and ensure a comprehensive review of the subject matter. For manuscript authors, it simplifies the literature review process, aids in source management, and ensures the inclusion of pertinent and up-to-date references, thereby enriching the quality and relevance of their academic writings (Table 5) [33-36].

**Table 5:** Applications, specific advantages, and disadvantages of the use of Research Rabbit in medical research and writing

S. No.	Applications
1.	Literature review assistance: Research Rabbit is an AI-powered literature review tool that simplifies the process of conducting literature reviews for researchers
2.	Visualizing papers: researchers can visualize papers in Research Rabbit, enabling them to explore and navigate the literature "forest" seamlessly, making connections between relevant papers
3.	Author network discovery: the tool offers author network discovery, allowing researchers to identify and explore connections between authors based on their publications and co-authorships
4.	Collection and collaboration: users can create collections of papers and collaborate with others, making it easy to work together on literature review projects and share research findings
5.	Personalized recommendations: Research Rabbit provides personalized recommendations based on the papers added to collections, improving over time to better suit the researcher's preferences
6.	Interactive visualizations: researchers can use interactive visualizations, such as graphs, to dive deeper into connections between papers and identify new research directions
7.	Integration with citation managers: the tool allows importing papers from popular citation managers like Mendeley, Zotero, and EndNote, streamlining the process of organizing relevant papers for review
8.	AI-based literature mapping: Research Rabbit uses AI to assist researchers in navigating the complex process of literature review, finding relevant papers, and discovering new avenues for exploration
9.	Free for researchers: the tool is available for free to researchers, offering an accessible and efficient way to streamline the literature review process and enhance research workflows
10.	Citation-based literature mapping: optimizes time searching for references for essays, minor projects, or literature reviews
Advantages	
1.	Enhanced literature writing: Research Rabbit helps in combining multiple related articles and integrating them into a coherent paper, making literature writing more effective
2.	Seed papers usage: initiates search with one or more seed papers to find relevant literature
4.	Personalized/dynamic recommendations: Research Rabbit provides personalized digests and recommendations related to researchers' collections, keeping them updated on the latest relevant papers
5.	Visualizations and networks: the tool offers interactive visualizations of paper networks and author connections, facilitating exploration and a deeper understanding of the research landscape
6.	Collaborative features: Research Rabbit allows collaboration on collections and provides a platform for researchers to share and discuss their findings with peers
9.	Reduces searching friction/single interface Research support: simplifies the process of finding relevant papers, avoiding cumbersome switching between databases
10.	Free of charge: no cost involved in using the tool, making it accessible to everyone
11.	Support for multiple projects: allows for the creation and management of multiple collections for different research projects
12.	Public source scanning: scans for publicly available papers based on similarities
Disadvantages	
1.	Limited source types: primarily finds scholarly papers, potentially missing out on other types of sources like books
2.	Necessity of seed papers: requires seed papers to start the search, which might be a limitation if starting research from scratch without any leads.
3.	Potential overwhelms with options: the variety of visualization and exploration options might overwhelm new users or those preferring simpler platforms
4.	Navigation challenges: the platform presents many buttons and a high volume of information, which can make it difficult for new users to know where to start
5.	Linera exploration limitation: limits users to a single linear path of exploration without the option to save or mark previously explored paths unless added to a user’s collection.
6.	Author disambiguation issues: problems with author disambiguation may result in a single author appearing as multiple nodes in a collaborative network

SciSpace

SciSpace leverages advanced technology to streamline manuscript writing and research, providing a comprehensive platform that integrates seamlessly with journal and conference formatting guidelines. Its core technology facilitates easy management of citations by integrating with reference management tools like Mendeley, Zotero, and EndNote, simplifying the bibliography process. Real-time collaboration features are powered by sophisticated backend algorithms that enable multiple authors to work on documents simultaneously, enhancing teamwork efficiency. Additionally, SciSpace's technology supports direct submissions to journals and conferences and offers compatibility with various file formats, including LaTeX, Word, and PDF. This blend of cutting-edge technology and user-friendly design significantly boosts productivity and simplifies the publication journey for researchers (Table 6) [37-40].

**Table 6:** Applications, specific advantages, and disadvantages of the use of SciSpace in medical research and writing

S. No.	Applications
1.	Verified journal templates: access to over 100,000 verified journal templates to auto-format research manuscripts, ensuring submission-readiness with one-click formatting
2.	Collaborative editing: collaboratively edit and review research manuscripts in Word format with peers, streamlining the review and revision process
3.	Visual elements integration: easily add visual elements like images and graphs to research articles without disrupting the formatting of the manuscript
4.	Language quality enhancement: improve the language quality of the manuscript with built-in grammar and spell-check tools, ensuring a professional and error-free writing style
5.	LaTeX function library: access a library of LaTeX functions to efficiently include mathematical expressions and scientific symbols in research articles
6.	Contextual summaries: use the AI tool to highlight confusing text, math, and tables in research papers, and get contextual summaries and explanations for better understanding
7.	Follow-up questions: ask follow-up questions to dive deeper into a topic, removing doubts and clarifying complex concepts while reading scientific articles
8.	Preset questions: find quick summaries, future works, and related papers to scientific articles, facilitating faster literature reviews and information gathering
9.	Notes and saving: save notes of interesting Copilot responses or parts of the PDF to keep essential information at your fingertips for future reference
10.	Powerful editor: SciSpace editor equipped with in-built tools for research actions, including adding and citing references, creating scientific expressions, cross-referencing, and more
11.	Collaboration and version control: seamlessly collaborate with authors and reviewers, track changes, and ensure efficient version control during the research writing process
12.	Automated data analysis: SciSpace Copilot uses AI to analyze scientific data, and identify patterns, and relationships, assisting researchers in making better decisions during data analysis
13.	Insights and suggestions: generate insights and suggestions based on analyzed data, helping researchers identify trends and areas of research that might not be immediately apparent
14.	Access to full-text PDFs: SciSpace Discover offers unrestricted access to relevant peer-reviewed articles in full-text PDFs, aiding in literature reviews and enhancing research knowledge discovery
15.	Personalized recommendations: get personalized article suggestions and stay up-to-date with trending topics to explore relevant papers and contribute to the research field effectively
16.	Insight generation: identifies patterns, trends, and relationships in scientific data, offering new hypotheses and underexplored research areas
Advantages	
1.	AI-powered assistance: SciSpace offers an AI-powered research assistant that helps researchers comprehend complex text, equations, tables, and diagrams found in research papers
2.	Time-saving: the AI tool enables users to quickly understand and decode complex content, reducing the time spent on comprehending research papers
3.	Simplified literature review: SciSpace's AI-powered literature review feature allows users to search for relevant papers, authors, topics, and more without relying solely on specific keywords
4.	Intelligent/automated citation generator: SciSpace provides an intelligent citation generator that supports various citation styles, allowing researchers to quickly generate accurate citations for their research articles
5.	Access to full-text PDFs: SciSpace provides unrestricted access to full-text PDFs, allowing researchers to easily access and review relevant research articles
6.	Personalized recommendations: SciSpace offers personalized recommendations for popular papers, topics, and conferences, helping researchers discover relevant content and stay updated in their field
7.	Direct manuscript submission: allows direct submission of manuscripts with appropriate formatting
8.	Plagiarism checking: integrates with Turnitin to ensure content integrity
9.	Enhanced decision-making: provides insights that help researchers make better-informed decisions regarding their projects
10.	Facilitates interdisciplinary research: supports collaboration across different scientific disciplines, addressing complex research questions
11.	Adaptive learning: uses ML to adapt recommendations based on the researchers’ behavior and preferences
12	User-friendly interface: designed to be accessible to researchers with varying levels of technical expertise
13	Real-time explanations and answers while reading research papers
Disadvantages	
1.	Limited free version: the basic plan is limited in features, and significant functionalities are reserved for the premium plan

Litmaps

Literature mapping is a crucial step in conducting a thorough literature review for academic research. It involves the organization and visualization of existing research to understand connections, identify gaps, and spot emerging trends. Literature mapping tools, leveraging AI and ML, have become invaluable in this process, simplifying the task of sifting through expansive bodies of work. These tools help researchers by creating visual representations of research landscapes, facilitating a more efficient review process. Among the top recommended tools are Connected Papers for one-click visualizations, Inciteful for iterative analysis with multiple seed papers, and Litmaps for overlapping visual maps and exploration features. Such tools are game-changers in academic writing, helping to structure reviews systematically and cohesively. Building on the significance of literature mapping in academic research, we will delve into Litmaps, a tool that stands out for its unique features in visualizing and expanding the horizons of literature review.

Litmaps is an intuitive, AI-enhanced research tool designed to assist scholars and researchers in mapping out the literature landscape of their specific field of study. It offers a dynamic, visual approach to understanding the relationships between different research articles, making it easier to track the development of theories and discover new connections within vast amounts of academic work. Litmaps simplifies the literature review process by enabling users to organize references in a way that reveals trends, gaps, and the most influential work in their area. This tool is particularly useful for constructing a comprehensive and well-structured literature review, ensuring that researchers are fully informed about the current state of knowledge and can position their work within the broader scholarly dialogue. With Litmaps, creating a thorough foundation for any academic project becomes more manageable and insightful (Table 7) [41-43].

**Table 7:** Applications, specific advantages, and disadvantages of the use of Litmaps in medical research and writing

S. No.	Applications
1.	Literature mapping: Litmaps offers literature mapping services that allow researchers to create interactive literature maps for different research topics
2.	Citation network visualization: the tool generates citation networks, providing visualizations of the literature cited by a research paper, enabling researchers to analyze development, subject areas, and impacts
3.	Seed paper exploration: researchers can input a seed paper, and Litmaps generates maps of the most relevant articles related to the seed paper, aiding in the exploration of related research articles
4.	Automated citation searching: utilizing automated citation searching, Litmaps helps researchers discover academic papers that are relevant to their research topics, streamlining the process of finding relevant data
5.	Visualization and tracing: the tool visualizes search results in Litmap, showcasing the relationships between articles through citation tracing, facilitating insights into literature and connections between papers
6.	Collaboration and sharing: researchers can collaborate with colleagues, share their Litmaps on social media, and export them to showcase their work, enhancing collaboration and knowledge sharing
7.	Literature review assistance: Litmaps assists researchers in streamlining the literature review process, helping them find relevant articles accurately and efficiently
Advantages	
1.	Visual representation: Litmaps creates interactive literature maps that visually display the articles relevant to your research topics, allowing for a better understanding of the connections between papers
2.	Insightful analysis: Litmaps allow researchers to gain insights into the literature on their topics by visualizing connections between papers based on similarity or citation relations
3.	Enhanced discovery: Litmaps enables researchers to discover papers they may not have found otherwise, expanding their knowledge and potentially uncovering new research directions
4.	Integration with reference managers: Litmaps offers the ability to link with reference managers, allowing researchers to seamlessly connect their existing reference collections with the application
5.	Organization and project management: Litmaps provides tools for organizing literature and managing research projects, aiding researchers in keeping track of key articles and project states
Disadvantages	
1.	Limited information: the available information about Litmaps is limited, making it difficult to provide a comprehensive list of advantages and disadvantages specifically for this application
2.	Dependency on citation network: the effectiveness of Litmaps is dependent on the citation network and the availability and accuracy of citation data
3.	Accessibility and availability: the accessibility and availability of Litmaps, including any subscription fees or access restrictions, may impact its usability for researchers
4.	Reliance on data quality: the accuracy and completeness of the literature database used by Litmaps can affect the reliability and relevance of the results
5.	Lack of collaboration features: Litmaps currently lacks collaboration features, which may limit its usability for collaborative research projects

QuillBot

QuillBot is an AI-driven writing assistant tailored for enhancing academic writing, offering features like paraphrasing to bypass plagiarism, suggesting synonyms, and grammar checking. It's particularly beneficial for those where English is a second language, ensuring their manuscripts read clearly and professionally. Its summarization tool is crucial for condensing intricate research into clear summaries. Although advantageous for time-saving and language enhancement, QuillBot's paraphrasing may occasionally lead to less personalized writing. Thus, while it aids medical writing, it requires judicious use alongside the writer's expertise (Table 8) [44-46].

**Table 8:** Applications, specific advantages, and disadvantages of the use of QuillBot in medical research and writing

S. No.	Application
1.	Paraphrasing: helps in rephrasing sentences and paragraphs while retaining the original meaning, aiding in avoiding plagiarism and improving the readability of the research article
2.	Sentence expansion: expands short sentences or bullet points into longer, more detailed explanations, enhancing the depth and thoroughness of the research article
3.	Synonym selection: suggests synonyms for words or phrases, allowing researchers to vary their vocabulary and avoid repetition, thereby improving the clarity and richness of the article
4.	Grammar correction: detects and corrects grammatical errors, punctuation mistakes, and typos, ensuring that the research article adheres to standard language conventions
5.	Idea expansion: generates additional ideas or points related to the research topic, helping researchers explore different angles and perspectives, and enriching the content of the article
6.	Summarization: creates concise summaries of longer texts or articles, allowing researchers to condense complex information and integrate key points effectively into the research article
7.	Plagiarism detection: checks for potential instances of plagiarism by comparing the research article with a database of existing literature, ensuring academic integrity and originality
Advantages	
1.	Language support: QuillBot offers support for multiple languages, making it versatile for researchers working in diverse linguistic contexts
2.	Collaboration: the collaborative features of QuillBot enable multiple researchers to work on a document simultaneously, facilitating teamwork and allowing for real-time revisions and feedback
Disadvantages	
1.	The quality of paraphrasing, language writing support, and feedback may vary, potentially resulting in inaccuracies or awkwardly structured sentences that require careful review

Common advantages, disadvantages, and ethical considerations of AI-powered tools in research and medical writing

The use of AI-powered tools in manuscript writing introduces a set of common advantages, disadvantages, and ethical issues that merit careful consideration. On the positive side, these tools significantly enhance efficiency, providing writers with instant grammar corrections, style improvements, and even content suggestions, thereby streamlining the writing process and elevating the quality of manuscripts. They democratize access to high-quality writing assistance, potentially leveling the playing field for non-native English speakers and less experienced writers. However, the disadvantages include a potential overreliance on AI, which may diminish the writer's skills and critical thinking abilities. There's also the risk of homogenization of writing styles, as AI suggestions may lead to a convergence toward a narrow interpretation of "good" writing. Ethically, the use of AI in writing raises questions about originality and authorship, with concerns about the potential for plagiarism and the erosion of individual voices. Furthermore, data privacy issues emerge from the AI's learning process, which often involves analyzing vast datasets of existing texts, potentially including sensitive or proprietary information. Overall, while AI-powered tools offer transformative potential for manuscript writing, they necessitate a balanced approach to leverage their benefits while mitigating their drawbacks and ethical concerns (Table 9) [47-50].

**Table 9:** Advantages, disadvantages and ethical considerations common to the use of all kinds of AI-powered tools for research and medical writing

S. No.	Advantages
1.	Comprehensive research workspace: AI-powered tools offer a research workspace with multiple features, providing a centralized platform for various research-related tasks
2.	Enhanced acceptance rates: aims to improve manuscript acceptance rates through professional services
3.	Time-saving: AI-powered tools can help save time by providing quick responses, generating ideas, summarizing information, and creating outlines
4.	Access to information: AI-powered tools have access to a vast amount of internet text sources and data, making them capable of providing information on a wide range of topics
5.	Overcoming language barrier: AI-powered tools help in translating manuscripts and improving manuscript quality for publication in English medical journals
6.	Writing efficiency: AI-powered tools enhance the writing process by automating routine tasks
7.	Automation/reduction of manual work: AI-powered tools can automate parts of the research workflow, such as literature review, by finding relevant papers, summarizing key information, language checks, plagiarism checks, content formatting, extracting important details, and many more
8.	Enhanced productivity/scalability: Elicit's AI capabilities can enhance researchers' productivity by quickly providing relevant information and insights, and producing a large volume of work in a short span of time
9.	Streamlined research workflows: Elicit aims to streamline research workflows by providing AI-powered assistance in tasks like brainstorming, summarization, and text classification
10.	Integration with various tools for enhanced productivity
Disadvantages	
1.	Lack of critical judgment and limited human input: AI-powered tools lack the nuanced judgment, expertise, and creative instincts inherent to human authors, potentially affecting the depth and innovation in complex medical discourse
2.	Cost: the financial investment required can be prohibitive
3.	Lack of personal views: The generated content may lack the researcher’s personal voice and views
4.	Misinterpretation: AI models may sometimes misinterpret research questions
5.	Potential bias in research selection: there is a possibility of bias in the selection and interpretation of research papers by the AI algorithms
6.	Limitations in complex writing: AI tools may face challenges in handling complex scientific concepts and may not fully capture the nuances required for intricate research writing
7.	Accuracy and reliability: the accuracy and reliability of AI-generated insights, connections, and recommendations may vary, requiring researchers to verify and validate the information independently if the model is trained on unreliable sources
8.	Learning curve: researchers may need to invest time in learning how to effectively utilize Elicit and maximize benefits
9.	Using and providing text without quoting citations and often incorrect citations
10.	Tool limitations: various AI platforms may have specific limitations or restrictions based on subscription plans, access levels, or usage policies
Ethical issues	
1.	Privacy and data security: researchers should be mindful of data privacy and security concerns when using AI tools and ensure the protection of sensitive information raising concerns about patient confidentiality and data protection. Ensuring privacy and compliance with regulations like HIPAA is critical
2.	Lack of transparency: black-box nature of AI decision-making can make it challenging to understand how conclusions are drawn. This lack of transparency complicates efforts to identify and correct errors or biases in the generated content
3.	Dependence on metadata quality: there’s a risk of inheriting biases present in the training data, which could lead to skewed results as the effectiveness of recommendations heavily relies on the availability and accuracy of metadata, which can sometimes be erroneous
4.	Importance of values: research integrity is founded on honesty, transparency, openness, rigor, accountability, and objectivity. AI-generated text poses a challenge to these values, as AI cannot be held accountable. AI-generated text may be passed as original work
5.	Dependency on AI and stifling creativity: reliance on an AI research assistant may lead to reduced independent critical thinking and analysis by researchers
6.	Bias: AI models may perpetuate biases present in their training data, leading to discriminatory or offensive language in the generated text. This could result in content that reflects certain perspectives disproportionately, potentially impacting the fairness and inclusivity of medical literature
7.	Job displacement: the automation of writing tasks by AI could potentially displace human medical writers, editors, and other professionals, leading to economic disruption and loss of employment opportunities
8.	Authorship and dependence: the appropriate attribution of authorship when using AI-assisted writing tools is debated, as is the potential over-reliance on AI for content creation, which could lead to a lack of critical engagement and validation
9.	Plagiarism: AI models trained on extensive text from existing literature might unintentionally generate content that closely mirrors source material, raising issues of originality and intellectual property rights

Recommendations for effectively integrating AI tools into medical writing processes, including overcoming challenges, ethical considerations, training, and skills

Several critical challenges and ethical considerations arise in the pursuit of integrating AI into research and medical writing. One significant disadvantage is the potential lack of critical judgment by AI tools, which could lead to biases in the research process. To mitigate these issues, it is essential to implement strategies like increasing human oversight and conducting rigorous algorithm audits to ensure accuracy and fairness. Addressing financial concerns is also crucial and can be approached by exploring a variety of cost-effective AI options that can accommodate budgetary constraints. Additionally, enhancing AI tools with expert human reviews could address the limitations of AI in complex interpretative tasks. Upholding academic integrity, fostering a balance between AI assistance and the researcher's creative input, and ensuring the protection of data privacy are also critical. These measures not only ensure the responsible use of AI but also safeguard the standards of scholarly writing and contribute to the trustworthiness of scientific discourse (Table 10) [47-48].

**Table 10:** Suggestions and recommendations to tackle the disadvantages and ethical considerations of the use of AI in research and manuscript writing

Disadvantages and ethical issues	Suggestions and recommendations
Lack of critical judgment and limited human input	Implement a review system where AI-generated content is always overseen and refined by human experts
Cost	Explore various AI tool options to find cost-effective solutions or consider open-source alternatives
Lack of personal views	Encourage researchers to edit AI-generated content to reflect their personal insights and perspectives
Misinterpretation	Include rigorous validation checks for AI interpretations and employ subject matter experts for final review
Potential bias in research selection	Regularly update and audit AI algorithms to minimize bias and reflect a diverse range of research
Limitations in complex writing	Use AI tools for initial drafts and rely on human expertise for nuanced and complex sections
Accuracy and reliability	Cross-reference AI-generated information with trusted sources and use multiple tools for validation. Encourage a culture where AI and human expertise are seen as complementary
Learning curve	Provide training and user support for researchers to maximize the effective use of AI tools
Incorrect citations	Integrate robust citation verification tools and manual checks into the writing process
Tool limitations	Choose tools that best fit the research scope and requirements, and be aware of the limitations
Privacy and data security	Adhere to data protection policies and use secure platforms to ensure confidentiality and compliance
Lack of transparency	Demand transparency from AI providers and prefer tools that offer explainable AI features
Dependence on metadata quality	Curate high-quality training datasets and continually update metadata to reflect accurate information
Undermining academic integrity	Embed ethical principles into AI training and enforced standards for AI-assisted research. Implement advanced AI-driven plagiarism detection tools and revisit and strengthen the peer review process with the AI scrutiny phase
Dependency on AI and stifling creativity	Balance the use of AI with independent research activities to foster creativity and critical thinking
Bias	Regularly audit AI outputs for bias, implement unbiased training data, and apply ethical AI practices
Job displacement	Integrate AI as a tool to augment, not replace, human roles, focusing on collaborative synergy
Authorship and dependence	Establish clear guidelines for authorship that recognize both AI assistance and human intellectual contribution
Plagiarism	Use advanced plagiarism detection with AI to ensure originality. Train users in proper citation and responsible use of AI to avoid plagiarism

Future directions

As we peer into the horizon of AI in medical research and writing, it becomes clear that the trajectory is toward an ever-increasing integration of AI technologies. Guiding principles suggest a future where AI serves to augment human effort, adhering to ethical standards that uphold integrity, transparency, validity, and accountability in scientific publishing. Advancements are likely to continue in enhancing AI’s ability to draft and even potentially lead to autonomous manuscript generation, while human oversight will remain indispensable for maintaining ethical and quality standards in the medical field. In light of these developments, fostering collaboration between AI developers, researchers, and journal editors will be critical to creating tools that meet the diverse needs of the medical writing community [49].

The anticipated future is one of collaborative synergy, where AI aids in organizing information logically, enhancing the understanding of complex medical concepts, summarizing extensive research, and fine-tuning grammar and style. AI tools will continue to refine the precision and simplicity of medical communication, making it globally accessible through translation and ensuring consistent terminology across documents. This evolution will be paralleled by a greater focus on maintaining an objective tone, automating citation and reference formatting, and crafting approachable patient communications. As AI technologies evolve, ongoing education and training for researchers in the responsible use of AI will be paramount, ensuring that the balance between human expertise and machine assistance remains harmonious [50].

## Conclusions

The integration of AI technologies in medical writing has evolved significantly over the years, driven by advancements in NLP and ML. Initially, AI's role in word processing was limited to basic grammar checks and formatting, but with the advent of deep learning and neural networks, AI tools like ChatGPT and others now assist with content generation, summarization, and text enhancement.

Current AI-powered tools provide substantial benefits in streamlining medical writing tasks, improving productivity, and enhancing the quality of documents. These tools, such as ChatGPT and Google Bard, and specialized research tools like Elicit and Consensus, are valuable in various stages of the research and writing process. However, challenges remain, including concerns about over-reliance on AI, potential biases, homogenization of writing styles, and ethical considerations such as data privacy, originality, and authorship. These tools also require careful human oversight to mitigate these risks. To effectively integrate AI into medical writing, it is crucial to strike a balance between AI support and human judgment. Training in the ethical and responsible use of AI, along with algorithmic audits and financial considerations, can address the limitations and potential biases in AI-driven research processes. Maintaining academic integrity, protecting data privacy, and ensuring collaboration between AI developers, researchers, and editors will help develop more tailored and useful tools for the medical writing community.

Looking forward, AI technologies will continue to advance, potentially automating even more complex aspects of medical writing. Nevertheless, human oversight will remain indispensable to uphold the quality, accuracy, and ethical standards required in medical research and publishing. By fostering a collaborative synergy between AI tools and human expertise, the medical writing process can be further enhanced, making medical communication more precise, accessible, and efficient across the globe.
